# Iridium-Doped Nanosized Zn–Al Layered Double Hydroxides as Efficient Water Oxidation Catalysts

**DOI:** 10.1021/acsami.0c07925

**Published:** 2020-06-25

**Authors:** Lucia Fagiolari, Marzia Bini, Ferdinando Costantino, Giordano Gatto, A. Jeremy Kropf, Fabio Marmottini, Morena Nocchetti, Evan C. Wegener, Francesco Zaccaria, Massimiliano Delferro, Riccardo Vivani, Alceo Macchioni

**Affiliations:** † Department of Chemistry, Biology and Biotechnology, University of Perugia and CIRCC, Via Elce di Sotto, 8, I-06123 Perugia, Italy; ‡ Department of Pharmaceutical Sciences and CEMIN, University of Perugia, Via Fabretti 48, I-06123 Perugia, Italy; § Argonne National Laboratory, Lemont, Illinois 60439, United States

**Keywords:** layered double hydroxides, nanomaterials, water oxidation, iridium, oxygen evolution, doped materials

## Abstract

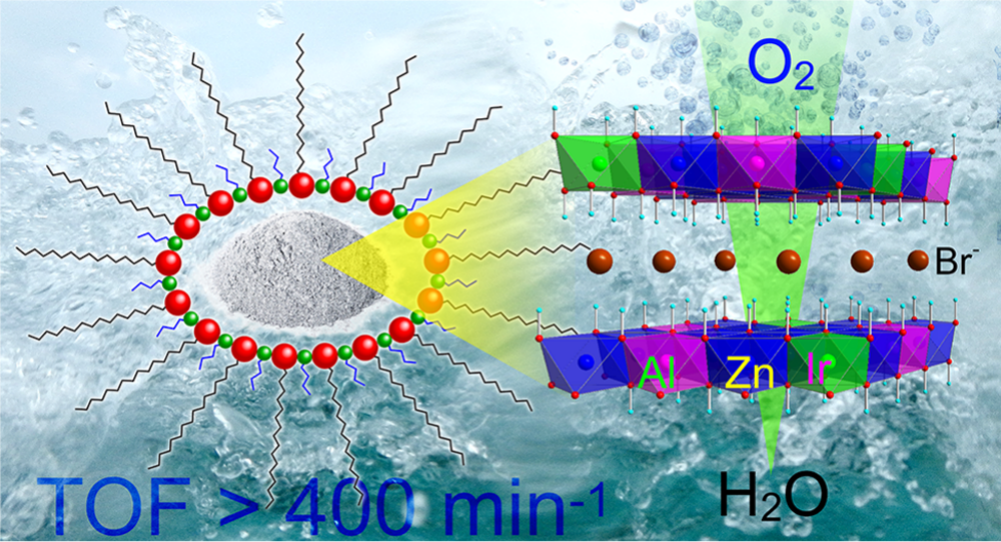

Layered double hydroxides (LDHs) are an ideal platform to host catalytic metal centers for water oxidation (WO) owing to the high accessibility of water to the interlayer region, which makes all centers potentially reachable and activated. Herein, we report the syntheses of three iridium-doped zinc–aluminum LDHs (Ir-LDHs) nanomaterials (**1–3**, with about 80 nm of planar size and a thickness of 8 nm as derived by field emission scanning electron microscopy and powder X-ray diffraction studies, respectively), carried out in the confined aqueous environment of reverse micelles, through a very simple and versatile procedure. These materials exhibit excellent catalytic performances in WO driven by NaIO_4_ at neutral pH and 25 °C, with an iridium content as low as 0.5 mol % (∼0.8 wt %), leading to quantitative oxygen yields (based on utilized NaIO_4_, turnover number up to ∼10,000). Nanomaterials **1–3** display the highest ever reported turnover frequency values (up to 402 min^–1^) for any heterogeneous and heterogenized catalyst, comparable only to those of the most efficient molecular iridium catalysts, tested under similar reaction conditions. The boost in activity can be traced to the increased surface area and pore volume (>5 times and 1 order of magnitude, respectively, higher than those of micrometric materials of size 0.3–1 μm) estimated for the nanosized particles, which guarantee higher noble metal accessibility. X-ray absorption spectroscopy (XAS) studies suggest that **1–3** nanomaterials, as-prepared and after catalysis, contain a mixture of isolated, single octahedral Ir(III) sites, with no evidence of Ir–Ir scattering from second-nearest neighbors, excluding the presence of IrO_2_ nanoparticles. The combination of the results obtained from XAS, elemental analysis, and ionic chromatography strongly suggests that iridium is embedded in the brucite-like structure of LDHs, having four hydroxyls and two chlorides as first neighbors. These results demonstrate that nanometric LDHs can be successfully exploited to engineer efficient WOCs, minimizing the amount of iridium used, consistent with the principle of the noble-metal atom economy.

## Introduction

The development of an efficient catalytic system for the oxidation of water (WO) to molecular oxygen is one of the most demanding challenges that the scientific community is facing nowadays. The large efforts lavished for the implementation of such a system are justified considering that WO, a potentially cheap and green source of electrons and protons, is a key component of any sustainable device for the generation of renewable fuels, driven by the sun.^1^

A plethora of water oxidation catalysts (WOCs) has been reported so far.^2−6^ Many efforts are currently made to develop earth-abundant metal WOCs, and notable progress has been achieved using, for instance, iron, cobalt, nickel, and copper.^2^ However, the systems exhibiting the best catalytic performances, in terms of activity and—especially—durability, are typically based on noble metals such as ruthenium and iridium.^3−6^ For this reason, many research groups are focusing on the optimization of WOCs in a “noble-metal atom economy” fashion, that is, trying to maximize the exploitation of the Ir/Ru centers while minimizing their content in the catalytic systems.^5,6^ “Noble-metal atom economy” can be pursued in the development of all three types of catalysts, namely molecular,^7−13^ heterogenized,^5,14−19^ and heterogenous^6,20−24^ systems.

Relating to the latter class, the classical strategy consists in tuning the particle size and/or morphology of noble metal oxides to maximize surface area and, therefore, the exposure of active centers.^24−27^ Alternatively, other successful approaches consist of using mixed metal oxides,^28−31^ depositing oxide nanoparticles^20,32,33^ or atomically dispersed metal centers^34,35^ on conductive supports, or doping layered inorganic materials of earth-abundant elements with small amounts of noble metals.^21,22,36−38^ An additional advantage of using mixed metal materials often lies in the possibility of having cooperative effects between redox active and nonactive centers,^39−41^ similar to what happens with Mn and Ca in the biological oxygen evolving complex.

Recently, we reported some Ir-doped Zn–Al-layered double hydroxides (Ir-LDHs) as highly efficient heterogeneous WOCs developed in “noble-metal atom economy”.^21,22^ These inorganic materials have the general formula [Zn_*x*_Al_*y*_Ir_*z*_(OH)_2_]Cl_*y*+*z*_·*m*H_2_O, in which *x* + *y* + *z* = 1, and the iridium centers typically account only for 1–3 mol % of the total metal content (corresponding to 1–5 wt % of the solid material). The characteristic brucite-like structure of LDHs makes these materials extremely versatile, finding broad and successful applications also for the oxygen evolution reaction.^42−44^ The positively charged layers, containing the divalent (Zn^2+^) and trivalent (Al^3+^) cations, are separated by interlayer regions where the anions (e.g., Cl^–^) are intercalated.^45^ In Ir-LDHs,^21,22^ some of the trivalent Al^3+^ cations are replaced by highly dispersed, dopant Ir^3+^ centers, which are exposed to the interlayer regions and, consequently, are easily accessible to water molecules.^42,46^

In WO driven by NaIO_4_ as a sacrificial oxidant (SO), Ir-LDHs of a micrometric dimension (about 0.3–1 μm) exhibit excellent turnover frequency (TOF) up to 113 min^–1^ and turnover numbers (TONs) > 11,900,^21^ which are comparable to those of some leading molecular WOCs.^5,7,10^ Furthermore, these heterogeneous systems are characterized by high stability and recyclability. Over eight successive runs, no leaching of noble metal to the liquid phase is detected by inductively coupled plasma–optical emission spectrometry (ICP–OES) analysis and no significant loss of activity is observed.^21^

Furthermore, when immobilized in carbon paste electrodes, Ir-LDHs were found to be suitable catalysts for electrochemical WO, with performances that exceed those of commercial IrO_2_.^22^ Monitoring the reaction by linear sweep voltammetry at 1 M KOH revealed that these systems catalyze WO with rather low overpotentials of 262–277 mV (at 10 mA/cm^2^) and quite high current densities of about 11–14 mA/cm^2^ (at 280 mV). Analogous Ru-LDHs appear to be initially more active, although far less durable. The replacement of bulk Zn with Mg centers was found to be detrimental for activity, pointing to some Zn/Ir cooperativity effects.^22^ These preliminary results in chemical and electrochemical WO appear very promising, especially considering that these Ir-LDHs are prepared following versatile and established synthetic procedures, with no need for complex strategies such as single atom electrodeposition,^37^ pulsed laser ablation,^47,48^ exfoliation,^49,50^ and fabrication of core–shell particles.^51,52^

**Figure 1:**
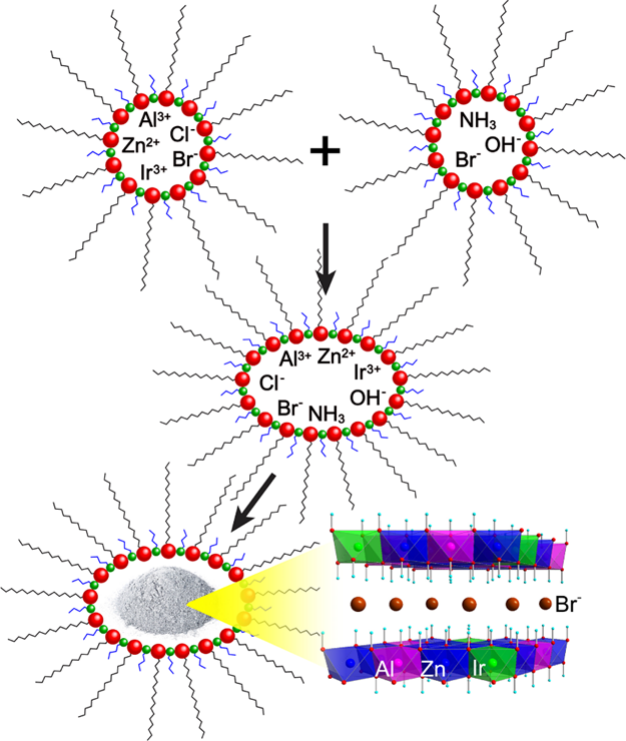
Pictorial representation of the preparation of nanometric Ir-LDH in the aqueous environment of reverse micelles.

In the present work, the possibility to enhance the performance of Ir-LDHs, by increasing the surface area of the particles to further improve the accessibility of the active centers, is explored. A series of nanosized Ir-LDHs were prepared by following the microemulsion synthetic strategy, in which the solid particles are grown in the aqueous media of reverse micelles (Figure 1).^53−55^ The materials were extensively characterized by a series of advanced spectroscopic techniques and tested in chemical (NaIO_4_-driven) WO.

## Results and Discussion

### Synthesis and Characterization

Three nanosized Ir-LDHs (**1–3**) were synthesized by reacting suitable amounts of Zn^2+^, Al^3+^, and Ir^3+^ aqueous solutions with NH_3_ as precipitating agent, within the confined environment of reverse micelles, in an *iso*-octane medium, and using cetyltrimethylammonium bromide (CTABr) and *n*-butanol as surfactants (Figure 1; see Experimental Part for details). This represents a practical and efficient strategy to tune particles’ sizes as their growth is limited by the controlled dimensions of the micelles.^53−55^ Previously reported Ir-LDHs of micrometric dimension (**4**),^22^ prepared by the urea method,^56,57^ were also considered in this study for comparative purposes.

The obtained samples have a general formula [Zn_*x*_Al_*y*_Ir_*z*_(OH)_2_]A_*y*+*z*_·*m*H_2_O in which *x* + *y* + *z* = 1 and A is the balancing anion, which is bromide for samples **1–3** obtained by the reverse micelle method because of the large excess of this ion in the intramicellar solution coming from the surfactant,^54,58^ whereas it is chloride for sample **4** obtained by the urea method.^22^ The value of *m* is generally close to 0.5. The metal loadings in samples **1–3** were determined by ICP–OES analysis and are reported in Table 1. It is interesting to outline that, for these nanometric samples, the Ir present in the reaction solution is almost quantitatively incorporated in the solid product (see Table S1), minimizing the noble metal loss during catalyst preparation. Conversely, for micrometric **4**, obtained by the urea method, less than 10% of the starting Ir is eventually incorporated into the final product.^21,22^ The possibility of tuning the stoichiometry of the solid by simply varying the salt concentration in solution, as well as limiting particle size growth by selecting the proper micelle system, makes this microemulsion method highly suitable for the straightforward and controlled synthesis of Ir-LDHs.

**Table 1:** Composition of [Zn_*x*_Al_*y*_Ir_*z*_(OH)_2_]A_*y*+*z*_·*m*H_2_O Samples As Determined by ICP–OES

sample	*x* (Zn)	*y* (Al)	*z* (Ir)
**1**	0.645	0.350	0.005
**2**	0.724	0.265	0.011
**3**	0.660	0.310	0.030
**4**^a^	0.647	0.349	0.004

a Microsized sample prepared with the urea procedure.

**Figure 2:**
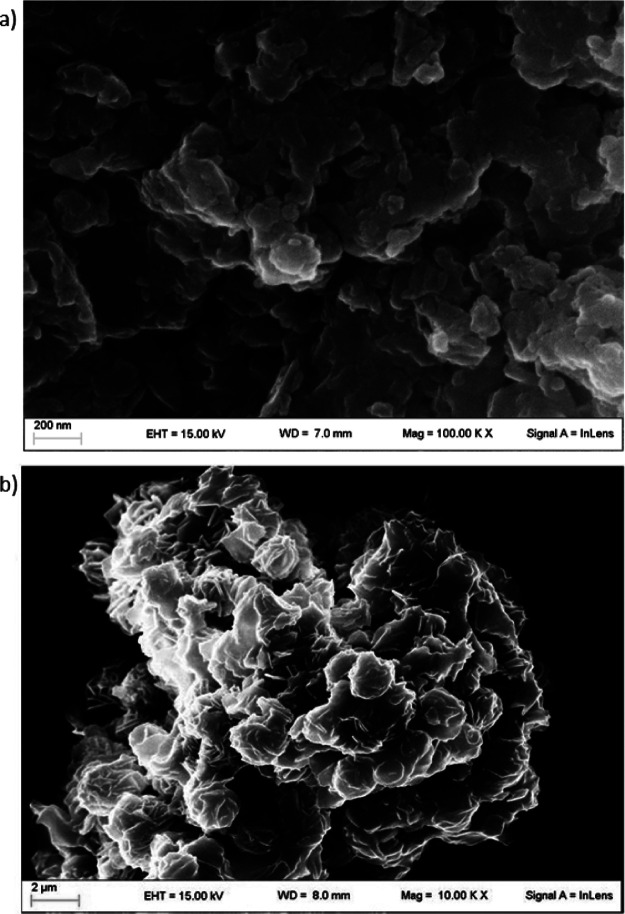
FE-SEM images of **2** (a) and **4** (b) showing the nanometric and micrometric size, respectively, of the catalytic particles.

Field emission scanning electron microscopy (FE-SEM) images confirm that **1–3** are constituted of highly aggregated nanometric platelets with an average planar size of about 80 nm (Figures 2a, S1, and S2), in line with literature data,^54,55,58^ which is about 1 order of magnitude smaller than that of sample **4** (average size of about 1 μm, Figure 2b).^22^

**Figure 3:**
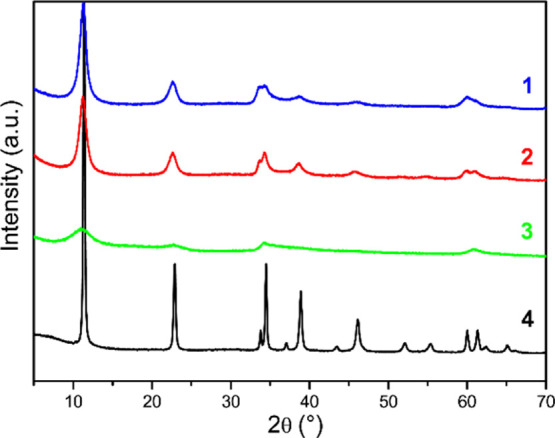
PXRD patterns of nanometric (**1–3**) and micrometric (**4**) Ir-LDHs.

Further morphological information can be derived by powder X-ray diffraction (PXRD) analysis (Figure 3). The pattern of **4** is typical of the ZnAl-LDH phase.^59,60^ Similar peaks are detected for **1–3**, although they are appreciably broader than those of **4**; the broadening of peaks increases from **1** to **3**, that is, with the increasing of Ir content. Two main features generally affect the broadening of diffraction peaks: crystallite size and microstrain, that is, lattice defects and distortions. The broadening of **1** and **2**, which contain a very small amount of Ir, can be easily interpreted only accounting for the particle size contribution: in particular, the application of the Scherrer equation to the (003) basal peak provides a thickness of about 8 nm, in agreement with previous data.^54,58^ On the contrary, sample **3**, having similar morphological features to **1** and **2**, shows an increased peak broadening very likely because of a relevant contribution arising from structural distortions due to the introduction of Ir. These distortions can hardly be justified solely based on the different sizes of the cations as the ionic radius of Ir^3+^ in octahedral coordination (0.68 Å) lies between that of Zn^2+^ and Al^3+^ (0.74 and 0.54 Å, respectively).^61^ On the other hand, ion chromatography (IC) analysis revealed the presence of chloride in Ir-LDHs, in a 1.8 molar ratio with respect to Ir, based on the combination of IC and ICP–OES results; X-ray absorption spectroscopy (XAS) evidenced that these chloride groups are bound to the iridium centers incorporated in the LDH phase (vide infra). It can be reasonably concluded that the presence of such chloride groups in the LDH phase, derived from the IrCl_3_ precursor, is responsible for the above-mentioned structural distortions that justify the large broadening of the PXRD patterns of nanometric samples.

Figure S3 shows N_2_ adsorption and desorption isotherms for all samples. Following the IUPAC classification,^62^ type IV isotherms were obtained. The specific surface area was calculated by the Brunauer–Emmett–Teller (BET) method^63^ and found to be considerably higher for nanometric (45–140 m^2^/g; Table S3) rather than for micrometric (10 m^2^/g) samples, as expected. Similarly, the pore volume, estimated by the Barrett–Joyner–Halenda (BJH) method,^64^ is larger for **1–3** (0.08–0.31 cm^3^/g) than for **4** (0.01 cm^3^/g). Sample **1** has a considerably higher surface area and mesopore volume, probably because of a particle packing of house-of-cards type, compared to samples **2** and **3** that, on the contrary, show a more compact packing (see Figures S1–S4). However, it should be noted that FE-SEM images and surface area measurements were performed on the dry solids, and it is well known that nanosized LDHs highly disperse when placed in water.^58^ Therefore, the different particle aggregations observed in the dry solids should not significantly influence the catalytic performances of the samples in aqueous solutions. Furthermore, the surface of these materials accessible to the solution may be much greater than that measured for the dry solid with the gas adsorption–desorption techniques.

**Figure 4:**
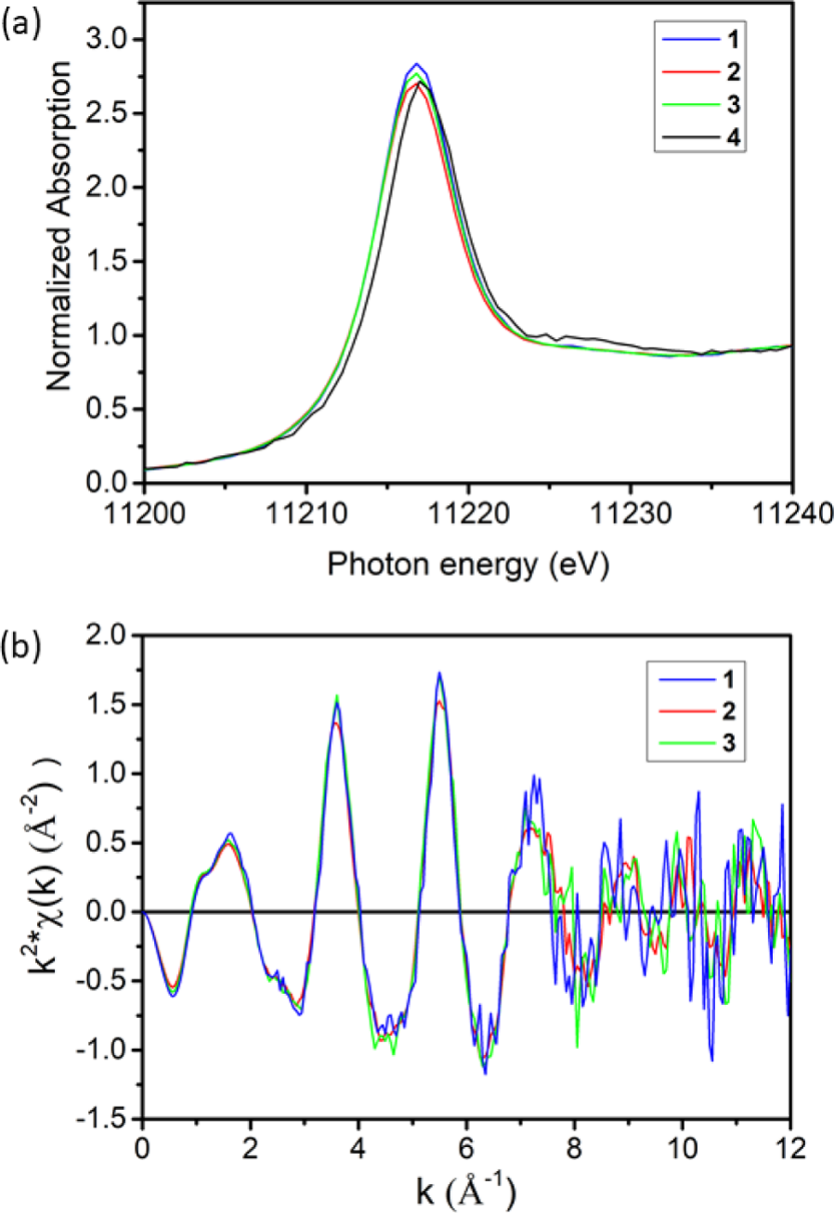
Iridium L_3_-edge XANES (a) spectra of **1–4** and *k*^2^-weighted EXAFS (b) spectra of **1–3**. Usable EXAFS of **4** could not be obtained because of a high level of noise (see also main text).

Iridium L_3_-edge X-ray absorption near-edge structure (XANES) energies of **1–3** are identical (11,214.5 eV) and very close to that of **4** (11,215.2 eV), as reported in Figure 4. These values are comparable to that of IrCl_3_ (11,214.3 eV), taken as reference, consistent with the hypothesis that the noble metal is in the +3 oxidation state in all the synthesized materials. The k^2^-weighted extended X-ray absorption fine structure (EXAFS) spectra of **1–3** are also similar (Figure 4), within the noise of the measurement, indicating that iridium has the same average structure in all four samples.

The magnitude of the Fourier transforms of the *k*^2^-weighted EXAFS shows a peak at 1.65 Å with a shoulder around 2.0 Å (phase uncorrected distances), as shown in Figure S4 for the representative case of **2**. This suggests that Ir centers have multiple types of nearest neighbors at different distances, in line with the poor quality of fits when using a single scattering path. A comparison of these spectra with those of reference species such as Ir(acac)_3_ and IrCl_3_ (acac = acetylacetonate; Figure S5) suggests that the peak at 1.65 Å is due to oxygen nearest-neighbors, whereas the shoulder arises from chloride nearest-neighbors. Consistently, phase-corrected Ir–O (2.02 Å) and Ir–Cl (2.33 Å) distances are very similar to those reported in the literature for *trans*-[Ir(H_2_O)_4_Cl_2_]^+^ (Ir–O = 2.04 Å and Ir–Cl = 2.35 Å).^65^ A two-scatterer fit was therefore performed using Ir–O and Ir–Cl paths and satisfactory results were obtained for **1–3** (Figure S4). Interestingly, despite the large excess of bromide anions in the samples, there is no evidence of Ir–Br scattering, suggesting that Br^–^ remains in the anionic layers of the Ir-LDHs. The EXAFS profiles could not be fitted accurately for micrometric **4** because of the high level of noise, but indications for the presence of a chloride fraction were obtained by XANES also for this sample (Figure 4).

The fitted coordination parameters are nearly identical for the three nanosized Ir-LDHs and summarized in Table 2. No evidence of Ir–Ir scattering from second-nearest neighbors is observed, as seen by the differences in the imaginary parts of the samples and the bulk oxide in the peaks between 2.3 and 4.0 Å (phase uncorrected distances; Figure S6). It appears therefore that all the iridium is in the LDHs structure and not as external nanoparticles. This is consistent with the aforementioned XANES results, which show that iridium is in the +3 oxidation state and not +4 like in IrO_2_.

**Table 2:** Ir L_3_-Edge XANES Energies and EXAFS Coordination Parameters for **1–3** and Some Reference Species^a^

entry	sample	edge energy (eV)	*S*_o_^2^	CN (scattering path)	*R* (Å)	σ^2^ (10^–3^ Å^2^)	*E*_o_ (eV)	*R*-factor
1	**1**	11,214.5	0.78	4.0 ± 0.2 (Ir–O)	2.02 ± 0.01	2.5	6.4 ± 1.3	*k*^1^: 0.002
				2.0 (Ir–Cl)	2.33	2.9	7.3	*k*^2^: 0.006
								*k*^3^: 0.018
2	**2**	11,214.5	0.78	4.1 ± 0.5 (Ir–O)	2.02 ± 0.02	2.5 ± 1.6	5.9 ± 2.3	*k*^1^: 0.008
				1.9 (Ir–Cl)	2.33	2.9	7.3	*k*^2^: 0.012
								*k*^3^: 0.019
3	**3**	11,214.5	0.78	4.0 ± 0.3 (Ir–O)	2.02 ± 0.02	2.5	6.0 ± 1.9	*k*^1^: 0.009
				2.0 (Ir–Cl)	2.33	2.9	7.3	*k*^2^: 0.013
								*k*^3^: 0.020
4	IrCl_3_·*x*H_2_O	11,214.3	0.78 ± 0.10	6 (Ir–Cl)	2.33 ± 0.01	2.9 ± 1.3	7.3 ± 1.3	*k*^1^: 0.015
								*k*^2^: 0.011
								*k*^3^: 0.010
5	Ir(acac)_3_	11,215.3	0.78 ± 0.09	6 (Ir–O)	2.00 ± 0.01	3.4 ± 1.6	4.8 ± 1.5	*k*^1^: 0.003
								*k*^2^: 0.006
								*k*^3^: 0.010
6	**2** (post-catalysis)	11,215.1	0.78	5.9 ± 0.5 (Ir–O)	1.98 ± 0.01	2.5 ± 1.1	6.3 ± 1.1	*k*^1^: 0.003
								*k*^2^: 0.006
								*k*^3^: 0.009
7	IrO_2_	11,215.5		6 (Ir–O)	1.99			

a Fitting ranges: **1–3** and IrCl_3_·H_2_O: Δ*k* = 3.0–11.7 Å^–1^, Δ*R* = 1.20–2.35 Å, and Ir(acac)_2_: Δ*k* = 3.0–11.7 Å^–1^, Δ*R* = 1.00–1.97 Å. *S*_o_^2^ = amplitude reduction factor, CN = coordination number, *R* = bond distance, σ^2^ = Debye–Waller factor, *E*_o_ = difference in threshold energy, *R*-factor = misfit between data and theory, *k* = photoelectron wave vector.

**Figure 5:**
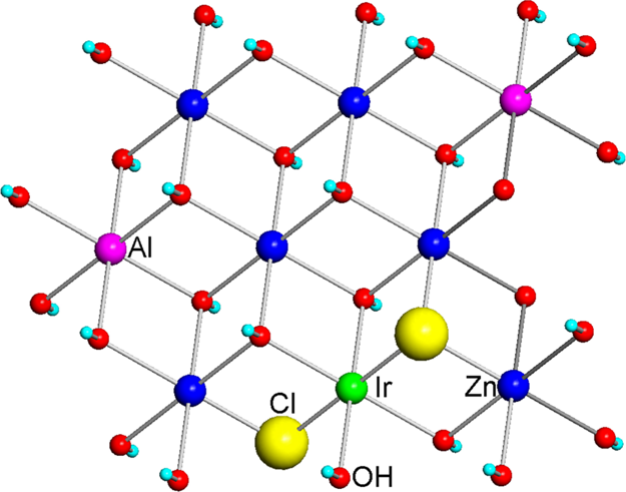
Sketch of the fragment of the brucite structure of Ir-doped Zn–Al LDHs showing the proposed network of Cl–Ir–Cl moiety interactions.

Thus, the results allow to conclude that Ir(III) ions are embedded in the LDH structure and are octahedrally coordinated by four oxygen and two chlorine anions (entries 1–3, Table 2). It is worth emphasizing that this Cl/Ir ratio is highly comparable to that estimated by combined IC and ICP–OES analysis (vide supra). Consequently, it appears more correct to express the formula of nanometric samples **1–3** as [Zn_*x*_Al_*y*_Ir_*z*_Cl_2*z*_(OH)_2–2*z*_]Br_*y*+*z*_·*m*H_2_O to indicate that the Cl–Ir–Cl moieties (probably in trans geometry)^65^ are integrated in the brucite-like structure of Zn–Al LDHs, with Cl^–^ (replacing OH^–^) acting as bridging ligands between Ir and Zn (Figure 5).

Additional XANES and EXAFS studies, performed on Ir-LDHs recovered after catalytic tests (vide infra), evidenced a variation of the average coordination environment and the disappearance of the shoulder at 2.0 Å (Figures S7 and S8; entry 2 *vs* 6, Table 2). The same is observed also upon extensively washing the Ir-LDHs in water at 80 °C (Figures S11 and S12). This suggests that all the Ir–Cl groups are transformed into Ir–O during catalysis, as further confirmed by IC. The fitting results (using six Ir–O bonds at 1.98 Å) are consistent with an octahedral coordination environment. A comparison of XANES, as well as k^2^-weighted EXAFS spectra, with those of the reference species, suggests that the octahedral Ir–O environment present in Ir-LDHs after catalysis is different from that of IrO_2_, despite the similarity in fitted Ir–O bond distance (1.98 Å for **2** after catalysis vs 1.99 Å for IrO_2_; Figures S9 and S10; entries 6 *vs* 7 Table 2). This indicates that the noble metal centers remain highly dispersed in the brucite-like scaffold of LDHs also during the catalysis.

### Catalytic Performance in WO

The catalytic performances of **1–4** toward WO were explored using sodium periodate as the SO. Experiments were carried out at pH 7 by phosphate buffer, monitoring oxygen evolution by differential manometry, according to benchmarked protocols.^7^ Catalytic experiments were carried out with iridium and NaIO_4_ concentrations ranging from 1 to 10 μM and from 5 to 40 mM, respectively (see Experimental Part for details). Based on duplicate experiments, and in line with previous measurements,^7,21^ a 10% error of on both TOF and TON is considered. Results are summarized in Table 3.

**Table 3:** Summary of Catalytic Results in NaIO_4_-Driven WO^a^

entry	[Ir] (μM)	[NaIO_4_] (mM)	TON	TOF (min^–1^)	yield^b^ (%)
**1**					
1	1	20	9470 ± 947	237 ± 24	95
2	2.5	20	4000 ± 400	220 ± 22	100
3	5	5	460 ± 46	84 ± 8	92
4	5	10	1000 ± 100	121 ± 12	100
5	5	20	1969 ± 197	211 ± 21	99
6	5	40	4000 ± 400	269 ± 27	100
7	10	20	953 ± 95	153 ± 15	95
**2**					
8	1	20	9690 ± 969	256 ± 27	97
9	2.5	20	3700 ± 370	207 ± 21	93
10	5	5	493 ± 49	75 ± 7	99
11	5	10	1000 ± 100	122 ± 12	100
12	5	20	1956 ± 196	163 ± 16	98
13	5	40	3604 ± 360	246 ± 25	90
14	10	20	974 ± 97	140 ± 14	97
**3**					
15	1	20	8730 ± 873	313 ± 31	87
16	2.5	20	3796 ± 380	256 ± 26	95
17	5	5	464 ± 46	139 ± 14	93
18	5	10	984 ± 98	189 ± 19	98
19	5	20	1960 ± 196	196 ± 20	98
20	5	40	3924 ± 392	402 ± 40	98
21	10	20	969 ± 97	205 ± 20	97
**4**					
22	1	20	9499 ± 950	142 ± 14	95
23	2.5	20	3927 ± 398	102 ± 10	98
24	5	5	500 ± 50	37 ± 4	100
25	5	10	1000 ± 100	53 ± 5	100
26	5	20	1985 ± 198	62 ± 6	99
27	5	40	3907 ± 391	98 ± 10	98
28	10	20	994 ± 99	73 ± 7	99

a Experimental conditions: 25 °C, 5 mL H_2_O, pH 7 by phosphate buffer.

b Estimated with respect to utilized NaIO_4_. A 10% uncertainty on both TOF and TON assumed based on duplicate experiments [for instance, at [Ir] = 5 μM and [NaIO_4_] = 20 mM, TON = 1963–1975 (**1**), 1944–1968 (**2**), 1920–2000 (**3**), 1970–2000 (**4**) and TOF = 207–215 (**1**), 161–164 (**2**), 193–199 (**3**), 57–67 (**4**) min^–1^] and in line with previous measurements.^7,21^

**Figure 6:**
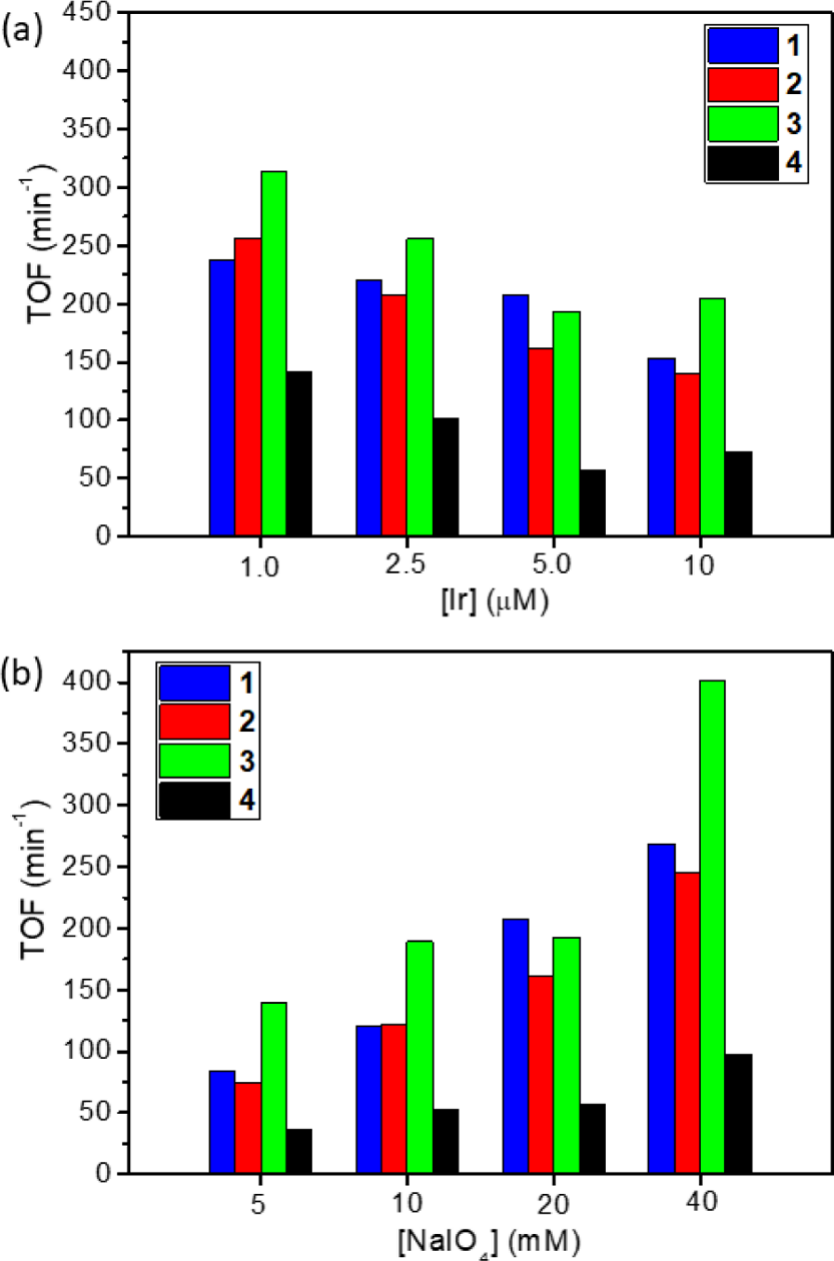
Comparison of TOFs for catalysts **1–4** at different iridium (a) or SO (b) concentrations (pH 7 by phosphate buffer, [NaIO_4_] = 20 mM).

For all samples, the developed O_2_ is almost quantitative and limited only by the amount of SO used (Table 3). TOF values are remarkably high for all Ir-LDHs and comparable to those of some leading molecular systems.^7^ The good performance of micrometric **4** is comparable to that of analogous systems reported in the literature, with TOFs in the range of 37–141 min^–1^.^21^ Gratifyingly, nanosized Ir-LDHs **1–3** are even more active and exhibit 2–4 times higher TOFs under every set of experimental conditions explored (Table 3 and Figure 6). In particular, catalysts **1** and **2** show comparable activity (84–269 and 75–256 min^–1^, respectively; entries 1–14, Table 3), whereas catalyst **3** exhibits the best overall performance, with TOFs ranging from 139 to 402 min^–1^ (entries 15–21; Table 3). Note that undoped LDHs of the type studied here are known to be inactive in WO under these experimental conditions.^21^

**Figure 7:**
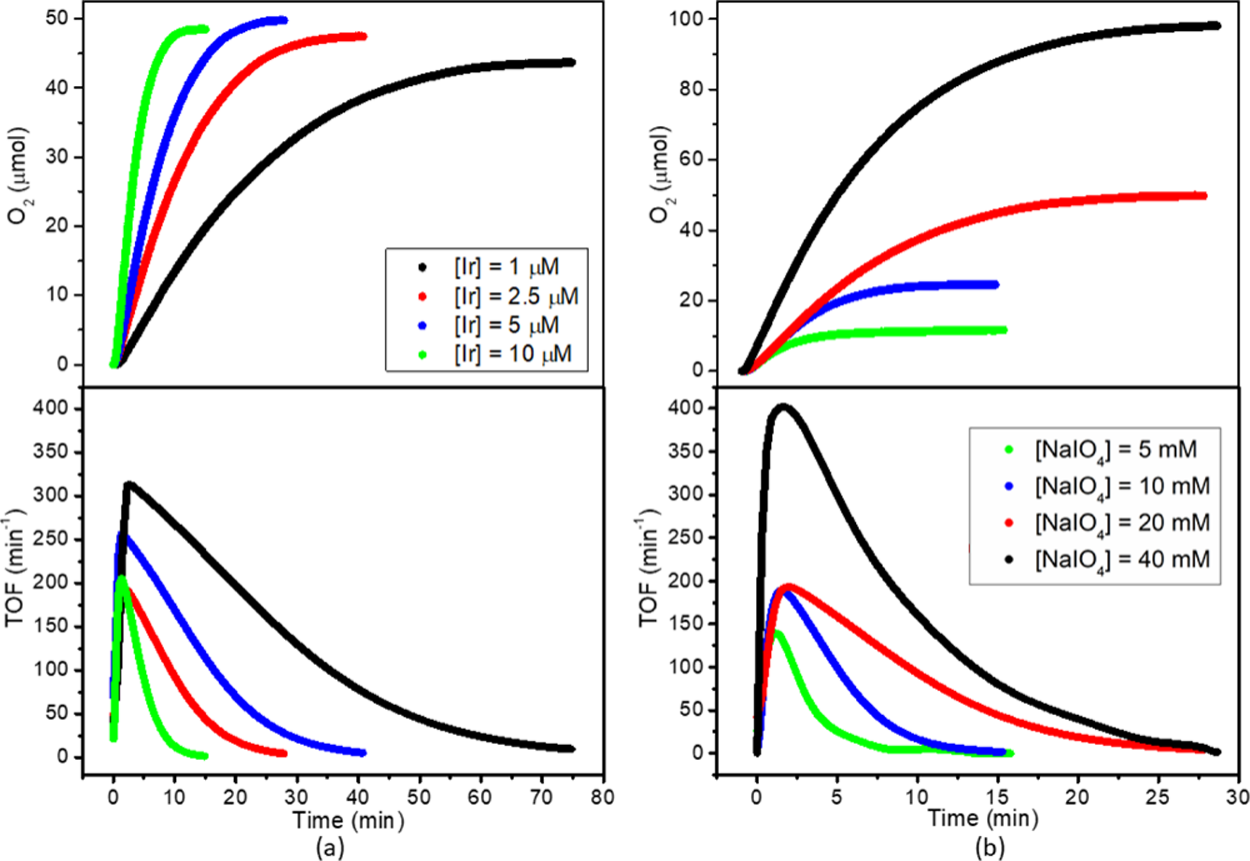
Kinetic profiles for WO by catalyst **3** (pH 7 by phosphate buffer, 25 °C) at different catalyst (a) and NaIO_4_ (b) concentrations.

Profiles of evolved O_2_ and TOF versus time are reported in Figure 7 for the representative case of **3**, while analogous graphs for **1**, **2**, and **4** are reported in the Supporting Information. It can be seen that the time necessary to obtain the highest TOF with **3** is ≤5 min, which is slightly smaller than that of catalysts **1** (∼5 min, Figure S13), **2** (≤10 min, Figure S14), and especially **4** (up to 18 min, Figure S15).

The activity appears to be dependent on both catalyst and SO concentration, reaching the maximum at low [Ir] and high [NaIO_4_] (Figure 6). Experiments at different concentrations allowed to estimate the reaction orders from the log–log plots reported in Figures S16 and S17. The reaction order in the catalyst (Figure S16) is found to be approximately 0.8 for nanometric **1–3**, which is slightly higher than that estimated here (0.7) and in previous reports (0.6)^21^ for micrometric Ir-LDHs. Although no definitive conclusions can be drawn based on such small (but appreciable) differences, the higher order observed for nanometric catalysts might be due to a higher fraction of active Ir centers with respect to micrometric systems as a consequence of the increased surface area, enhancing noble metal accessibility for **1–3** (see also Figure S18). The reaction order with respect to IO_4_^–^ was estimated to be 0.6 for **1** and **2**, 0.5 for **3**, and 0.4 for **4** (Figure S17). These broken values are rather typical for both molecular^7^ and heterogeneous^21^ catalysts in NaIO_4_-driven WO.

**Figure 8:**
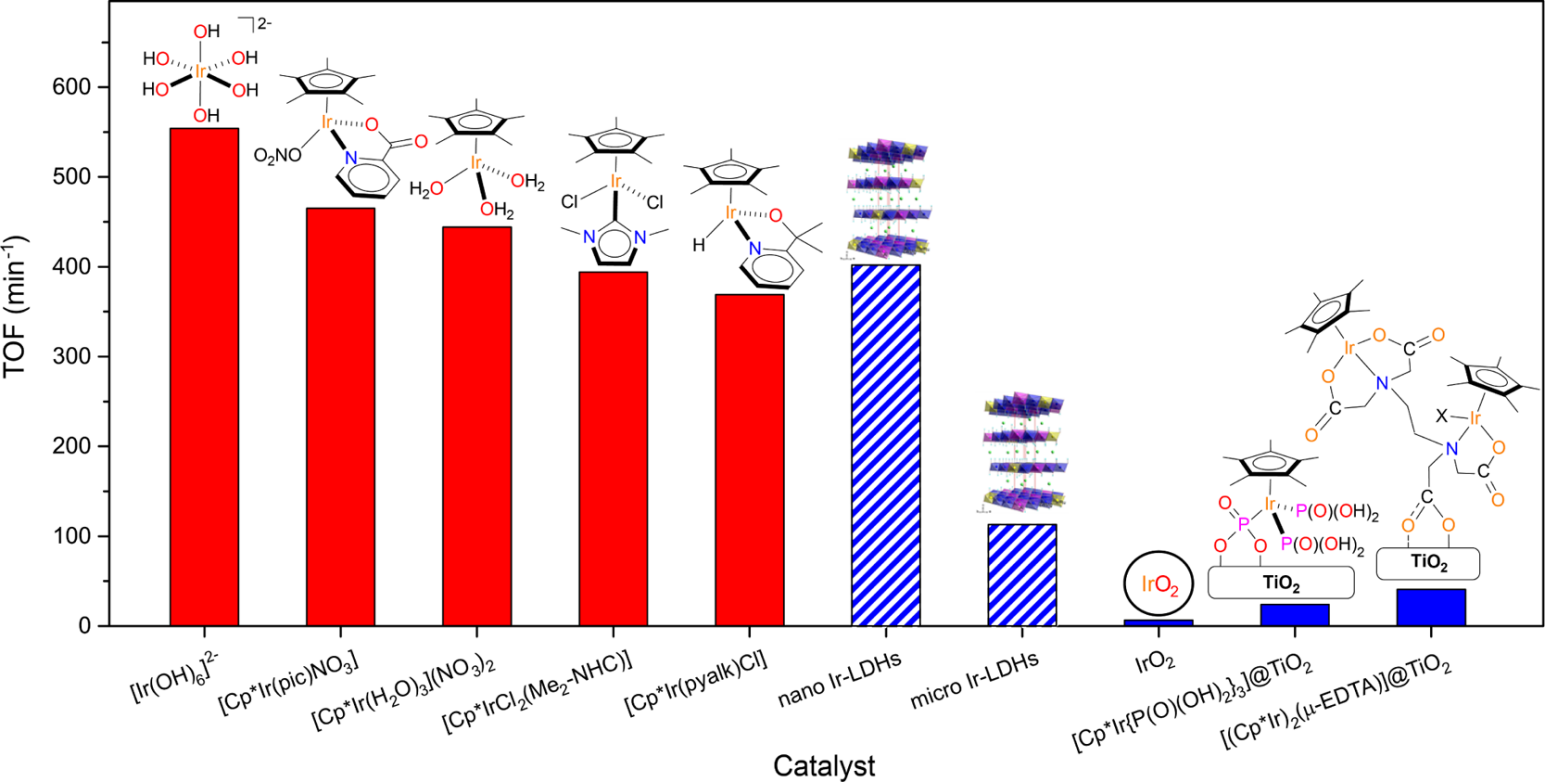
Graphical comparison of the maximum TOF reported in the literature for molecular (red) and heterogeneous/heterogenized (blue) catalysts with those observed here for micrometric and nanometric Ir-LDHs (striped blue) in NaIO_4_-driven WO.

Overall, the catalytic performances of **1–3** nanometric Ir-doped LDHs are remarkable for several reasons. First, the observed TOF values are comparable with those of the best molecular catalysts reported so far and much higher than those of heterogeneous and heterogenized catalysts (see Figure 8 for a graphical comparison of the representative examples discussed in the following).^7^ In a benchmarking study, we compared the catalytic activity of a number of well-known Ir-catalysts for WO driven by NaIO_4_ tested under the same experimental conditions.^7^ Catalysts exhibiting the highest TOF values were [Ir(OH)_6_]^2–^ (TOF up to 554 min^–1^) [Cp*Ir(pic)NO_3_] (TOF up to 465 min^–1^, pic = 2-pyridine-carboxylate), [Cp*Ir(H_2_O)_3_](NO_3_)_2_ (TOF up to 444 min^–1^), [Cp*IrCl_2_(Me_2_–NHC)] (TOF up to 394 min^–1^, Me_2_–NHC=*N*-dimethylimidazolin-2-ylidene), and [Cp*Ir(pyalk)Cl] (TOF up to 369 min^–1^, pyalk = 2-pyridine-isopropanoate). The TOF observed for **3** Ir-doped Zn–Al nanometric material is 402 min^–1^, significantly lower, but not that far, only than that of [Ir(OH)_6_]^2–^. A few heterogeneous catalysts have been tested in WO driven by NaIO_4_ and they show modest TOF values (e.g., TOF = 6.5 min^–1^ for IrO_2_^21^). Also, heterogenized catalysts exhibit TOF considerably lower than those of **1–3**. Fastest heterogenized catalysts are [Cp*Ir{P(O)(OH)_2_}_3_]@TiO_2_ (TOF up to 23.7 min^–1^)^19^ and [(Cp*Ir)_2_(μ-κ^3^-O,N,O-EDTA)]@TiO_2_ (TOF up to 41 min^–1^).^66^ Second, **1–3** are completely inorganic catalysts, extremely robust also under the harsh conditions necessary for carrying out water oxidation, which do not show any sign of deactivation, always providing the maximum value of TON (approaching 10,000 herein), compatible with the amount of NaIO_4_ used. The excellent recyclability of micrometric Ir-LDH has been widely proven in previous studies^21,22^ and further verified here for the representative nanomaterial **2** (Figure S19). Third, no leaching of iridium has been observed for Ir-doped LDH materials in general, probably because Ir is embedded in the brucite structure, as strongly suggested by XANES and EXAFS results herein reported.

## Conclusions

Nanometric Ir-doped Zn–Al LDHs are very promising WOCs, whose strength stems in having *diluted* and *easily accessible active metal centers*, embedded in the LDH structure, *which cannot aggregate into IrO*_*2*_*nanoparticles*. As for the dilution, which is extremely important considering the low abundance of iridium, herein we show that already 3.0 mol % mol is enough to reach top performances (TOF up to 402 min^–1^). This allows working according to the principle of “noble-metal atom economy”. The possibility of using Ir-diluted materials probably derives from having most of iridium centers reachable by water and the oxidant, owing to the nanometric dimensions of Ir-doped LDH materials, which causes a consistent enhancement of the surface area and by their implicit layered structure that allows substrate diffusion. The former explanation is confirmed by the observation that the performances of these systems largely exceed that of analogous micrometric systems (TOF up to 142 min^–1^) under all reaction conditions explored.

Furthermore, herein we show by XANES and EXAFS results that Ir^3+^ is embedded and perfectly integrated into the brucite structure of LDHs, probably isomorphically substituting Al^3+^, although it shows two chloride ligands in its first coordination sphere, which are replaced by oxygen atoms during catalysis. Furthermore, it was established that Ir is unable to diffuse and aggregate with other iridium cations possibly leading to IrO_2_, which would cause some loss of activity. TON is very high and only limited by the amount of SO used. The observed TOF up to 402 min^–1^ is very similar to that reported for the best Ir-molecular WOCs, with Ir-LDH offering the further advantage of being heterogeneous inorganic catalysts, whose stability under the harsh experimental conditions could be hard tested.

All these considerations therefore confirm the great potentialities of Ir-doped LDHs as heterogeneous WOCs developed in “noble-metal atom economy”, which can still be amenable to further significant improvement.

## Experimental Part

### Materials and Methods

AlCl_3_·6H_2_O, CTABr, *n*-butanol, *iso*-octane, NH_3_ 1.25 M in water, methanol, diethyl ether, fuming HNO_3_, NaIO_4_, Na_2_HPO_4_·7H_2_O, and NaH_2_PO_4_·H_2_O were purchased form Sigma-Aldrich. ZnCl_2_, IrCl_3_·3.7H_2_O, and urea were purchased from Carlo Erba, Alfa Aesar and PlusOne, respectively. All chemicals were used without any further purification, unless otherwise stated. Milli-Q deionized water was used for synthesis, ICP–OES, and catalytic measurements. For the synthesis, water was further decarbonated by prolonged boiling under nitrogen atmosphere. **4** was synthesized as reported in the literature.^22^

### Synthesis of Nanometric Ir-LDHs

Previously reported protocols were adapted for the synthesis of **1–3**.^53−55^ CTABr was used as the surfactant, *n*-butanol as the cosurfactant, and *iso*-octane as the oil phase. Two microemulsions, designated A and B, were prepared by adding 25.8 mL (18 mL of *iso*-octane and 7.8 mL of *n*-butanol) of a CTABr 0.66 M solution to (A) a water solution (6.75 mL) of ZnCl_2_, AlCl_3_, and IrCl_3_ (total concentration = 0.525 M) and (B) a NH_3_ 1.25 M water solution (see Table S2). Microemulsions A and B were mixed together under stirring at room temperature for 15 min. Instantaneously, the system became cloudy, after that it was aged for 20 h at 75 °C. Particles were recovered by centrifugation, washed with deionized water (2 × 13 mL), methanol (2 × 13 mL), and diethyl ether (2 × 13 mL), and dried at room temperature overnight.

### Characterization

PXRD patterns were collected on a PANalytical X’PERT PRO MPD diffractometer operating at 40 kV and 40 mA, with a step size of 0.017° 2θ and step scan rate of 20 s, using Cu Kα radiation and an X’Celerator detector. The morphology of the sample was investigated using a FEG LEO 1525 field emission scanning electron microscope. Metal analyses were performed using a Varian 700-ES series inductively coupled plasma–optical emission spectrometer by dissolving the powders with few drops of fuming HNO_3_ and then diluting with Milli-Q ultrapure water. Nitrogen adsorption–desorption isotherms were determined with a Micromeritics ASAP 2010 instrument at 77 K on samples outgassed overnight at 373 K. The specific surface area and mesopore volume were calculated by BET^63^ and BJH^67^ method, respectively. IC for chloride analysis was performed by a Dionex 500, after suspending the solid samples in 1 M Na_2_CO_3_ solution at 80 °C for 4 h.

XAS experiments were conducted on the bending magnet beamline of the Materials Research Collaborative Access Team (MRCAT) at the Advanced Photon Source, Argonne National Laboratory. Measurements were made in step-scan fluorescence mode using a four-element solid-state detector (Vortex ME4). Samples were prepared by diluting the LDH materials in a 50/50 mixture of boron nitride and PVP and then pressing the mixture into 7 mm wafers. XAS spectra were analyzed using the Demeter software suite.^68^ Standard procedures were used for spectra normalization and background subtraction. The oxidation state of Ir was determined by comparing the normalized XANES of the samples with those of known reference compounds: iridium(III) chloride hydrate, iridium(III) acetylacetonate, and iridium(IV) oxide. EXAFS coordination parameters were determined from simultaneous fits in *R*-space of the Fourier transform of the *k*^1^, *k*^2^, and *k*^3^-weighted EXAFS. Theoretical phase shift and backscattering amplitude fitting functions were calculated using the FEFF software.^69^*S*_0_^2^ was determined to be 0.78 from fits of Ir(acac)_3_ and IrCl_3_ in which the coordination numbers were fixed at the known value of the compounds. This value was then fixed in the fits of the samples. To reduce the number of free parameters during two-path fits of the samples, the bond distance, Debye–Waller factor, and energy shift parameters for Ir–Cl were fixed at the fitted values of the IrCl_3_ reference. Initial fits of the samples in which both the Ir–O and Ir–Cl coordination numbers were allowed to vary resulted in total coordination numbers of about 6, consistent with the oxide and chloride species each being octahedral. In the final fits, the total coordination number was constrained to a value of 6 to allow for better estimates of the fractions of each species present and a more consistent comparison between samples to be made. The fraction of each species present was estimated by dividing the fitted fractional coordination number by the total coordination number of the species.

### Chemical Water Oxidation

Catalytic experiments were performed in water at pH 7 by phosphate buffer, measuring the evolved oxygen in the gas phase with a Testo-521 differential manometer. In a typical catalytic run, a suitable amount Ir-LDHs was suspended in 5 mL of buffered water in the working cell, while an equal amount of neat water was loaded into the reference cell. Both reactors were closed with a rubber septum, connected to the manometer, kept at a constant temperature of 25 °C, and placed under stirring for 20 min. Acquisition was started. When a steady baseline was achieved, a solution of NaIO_4_ was injected into the working cell through a gas-tight syringe. Gas evolution was monitored by measuring the differential pressure between the reference and working cells. The powdery solid catalysts remained well dispersed in the solution throughout the experiments. The resulting pressure versus time profiles were fitted with the Peters and Baskin (PB) equation^70^ to suppress experimental oscillations and facilitate mathematical analysis. The derivative of the PB fits provided reliable reaction rate trends as a function of time. The reaction rate over catalyst concentration led to TOF. Further details on the derivation of kinetic profiles can be found in the literature.^7^

## Supporting Information Available

Additional synthetic, characterization, and catalytic details (PDF)

## Abbreviations

LDHs: layered double hydroxides
WO: water oxidation
WOCs: water oxidation catalysts
TON: turnover number
TOF: turnover frequency
SO: sacrificial oxidant
