# Supplementary material for: Iridium-Doped Nanosized Zn–Al Layered Double Hydroxides as Efficient Water Oxidation Catalysts
Source: ACS Appl Mater Interfaces. 2020 Jun 25;12(29):32736–45. doi: 10.1021/acsami.0c07925 (PMC8008397; doi:10.1021/acsami.0c07925)
Supplement: Supplementary file 1 — am0c07925_si_001.pdf [file am0c07925_si_001.pdf]

Supporting Information

Iridium-Doped Nanosized Zn–Al Layered Double Hydroxides as Efficient Water Oxidation Catalysts

Lucia Fagiolari,[a,†] Marzia Bini,[b] Ferdinando Costantino,[a] Giordano Gatto,[a] A. Jeremy Kropf,[c] Fabio Marmottini,[a] Morena Nocchetti,[b] Evan C. Wegener,[c] Francesco Zaccaria,[a] Massimiliano Delferro,*[c] Riccardo Vivani,*[b] Alceo Macchioni*[a]

[a]Department of Chemistry, Biology and Biotechnology, University of Perugia and CIRCC, Via Elce di Sotto, 8, I-06123 Perugia, Italy;
[b]Department of Pharmaceutical Sciences and CEMIN, University of Perugia, Via Fabretti 48, I- 06123 Perugia, Italy;
[c]Chemical Sciences and Engineering Division, Argonne National Laboratory, Argonne, Illinois 60439, United States of America

* Email for A. M.: alceo.macchioni@unipg.it
* Email for R. V.: riccardo.vivani@unipg.it
* Email for M. D.: delferro@anl.gov

Summary

- Synthetic details 2
- Characterization details 3
- Kinetic plots for $NaIO_4$-driven WO 10

- **Synthetic details**

Table S1. Comparison of molar composition of reaction solution and solid products relative to the synthesis of **1**-**4**.

Sample	Solution (mol%)			Solid (mol %)		
	Zn	Al	Ir	Zn	Al	Ir
**1**	69.6	30.0	0.4	64.5	35.0	0.5
**2**	70.0	29.0	1.0	72.4	26.5	1.1
**3**	67.0	30.5	2.5	66.0	31.0	3.0
**4**	69.7	25.6	4.7	64.7	34.9	0.4

Concentrations are expressed in mol% with respect to the total metal content.

Table S2. Composition of oil and aqueous phase for microemulsions used for the synthesis of **1**-**3**.

Ir-LDH	Microemulsion	Oil phase	Aqueous phase
**1**	A	6.25 g CTABr<br>7.8 ml n-butanol<br>18 ml isooctane	6.75 ml of 0.525 M salt solution (0.3654 M $ZnCl_2$, 0.1575 M $AlCl_3$•$6H_2O$, 2.1 mM $IrCl_3$)
	B	6.25 g CTABr<br>7.8 ml n-butanol<br>18 ml isooctane	6.75 ml of 1.25 M NH_3 solution
**2**	A	6.25 g CTABr<br>7.8 ml n-butanol<br>18 ml isooctane	6.75 ml of 0.525 M salt solution (0.3675 M $ZnCl_2$, 0.1522 M $AlCl_3$•$6H_2O$, 5.2 mM $IrCl_3$)
	B	6.25 g CTABr<br>7.8 ml n-butanol<br>18 ml isooctane	6.75 ml of 1.25 M NH_3 solution
**3**	A	6.25 g CTABr<br>7.8 ml n-butanol<br>18 ml isooctane	6.75 ml of 0.525 M salt solution (0.3502 M $ZnCl_2$, 0.1606 M $AlCl_3$•$6H_2O$, 0.0142 M $IrCl_3$)
	B	6.25 g CTABr<br>7.8 ml n-butanol<br>18 ml isooctane	6.75 ml of 1.25 M NH_3 solution

- **Characterization details**

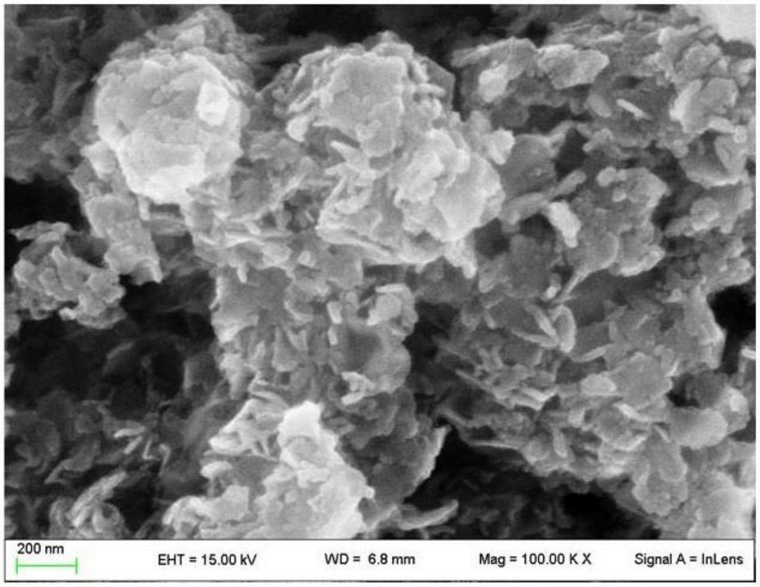

Figure S1. FE-SEM images of **1**.

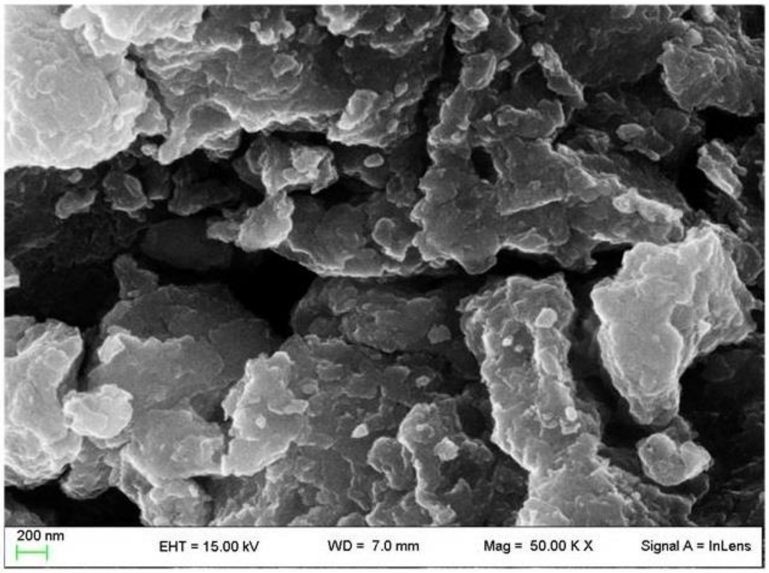

Figure S2. FE-SEM images of **3**.

Table S3. Surface area and mesopore volume obtained from N_2 adsorption and desorption measurements.

Sample	B.E.T.	BJH
	Surface area (m^2/g)	Mesopore volume (cm^3/g)
1	140	0.31
2	45	0.08
3	52	0.10
4	10	0.01

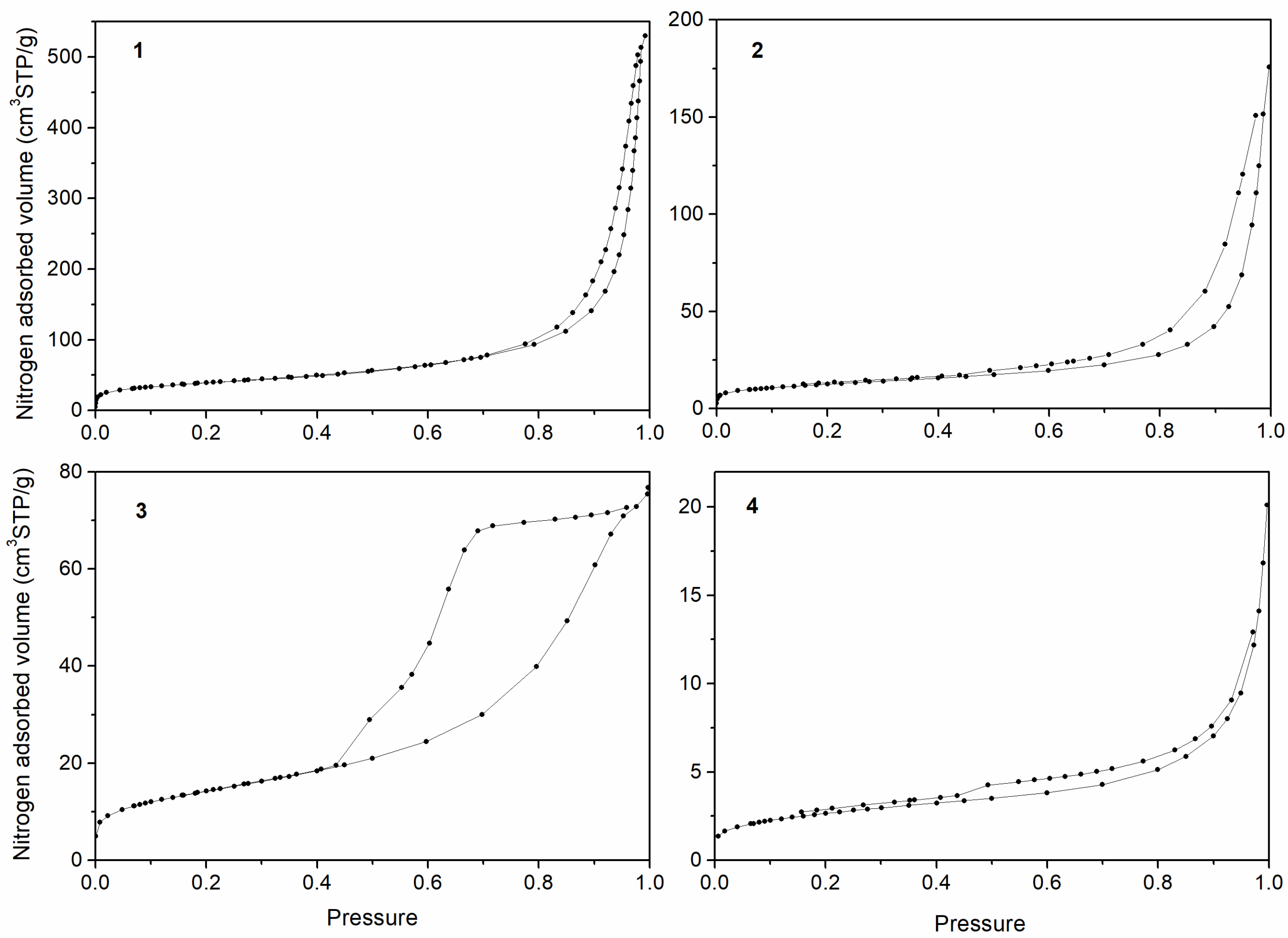

Figure S3. N_2 adsorption-desorption isotherm plots for **1**-**4**.

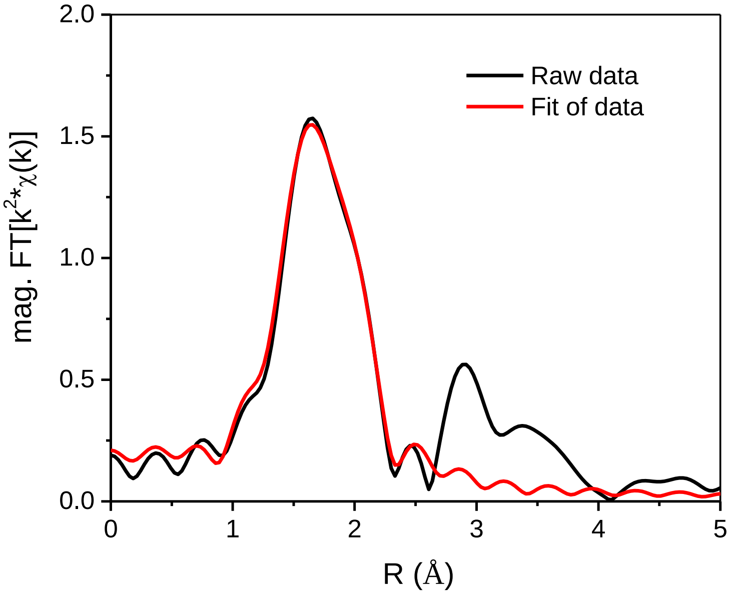

Figure S4. The magnitude of the Fourier transform of the k^2-weighted EXAFS of **2**: raw data (black) and two-path-fit of the first scattering shell (red).

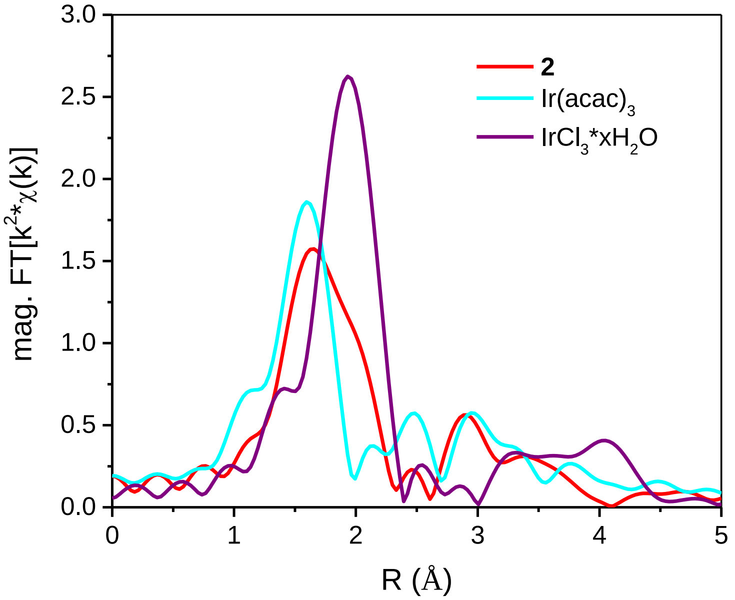

Figure S5. The magnitude of the Fourier transform of the k^2-weighted EXAFS of **2** and reference Ir(III) species like $Ir(acac)_3$ and $IrCl_3$.

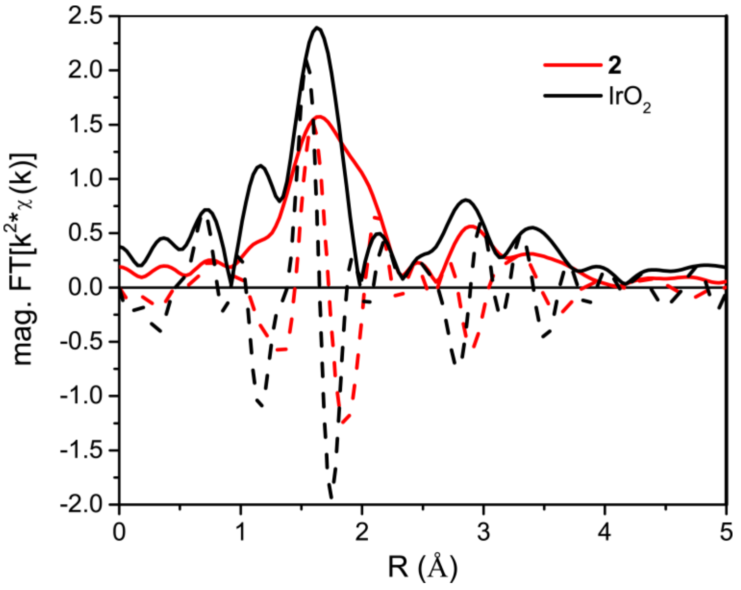

Figure S6. The magnitude (solid) and imaginary part (dashed) of the Fourier transform of the k^2-weighted EXAFS of **2** and IrO_2.

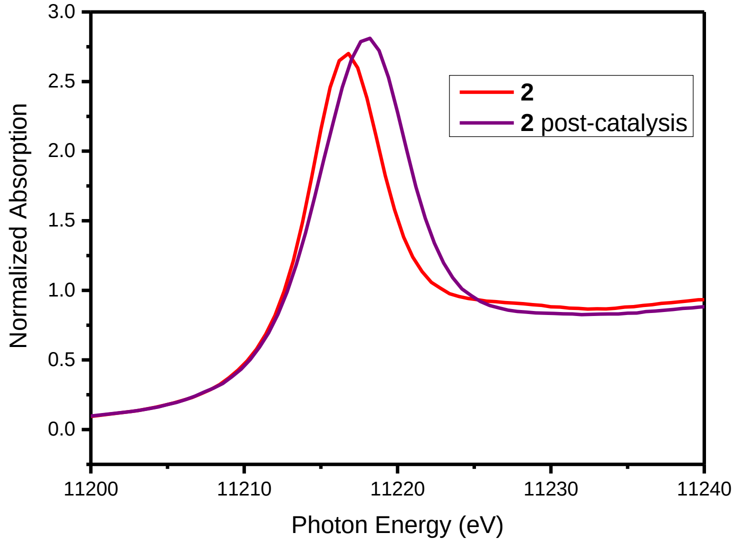

Figure S7. Ir L_3 edge XANES of **2** before and after catalytic run.

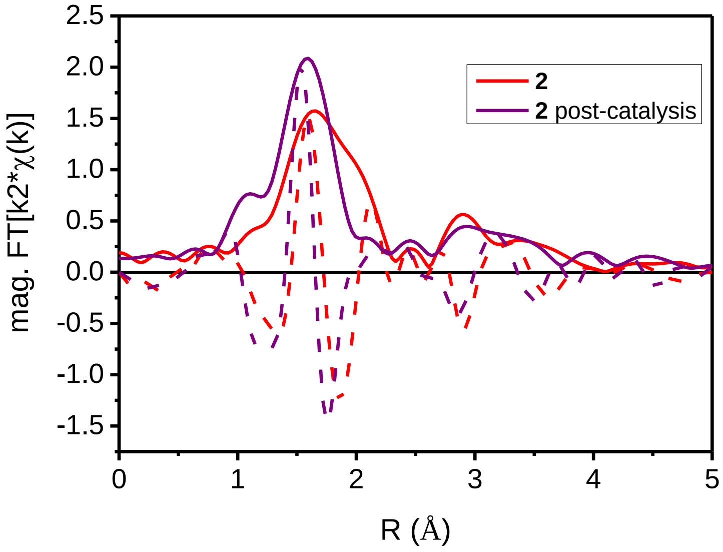

Figure S8. The magnitude (solid) and imaginary part (dashed) of the Fourier transform of the k^2-weighted EXAFS of **2** before and after catalytic run.

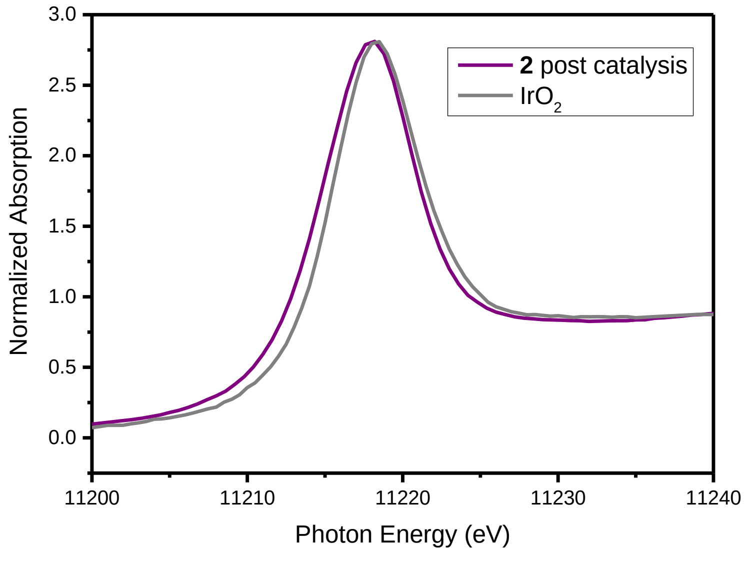

Figure S9. Ir L_3 edge XANES of **2** recovered after catalytic run in unbuffered water and reference IrO_2.

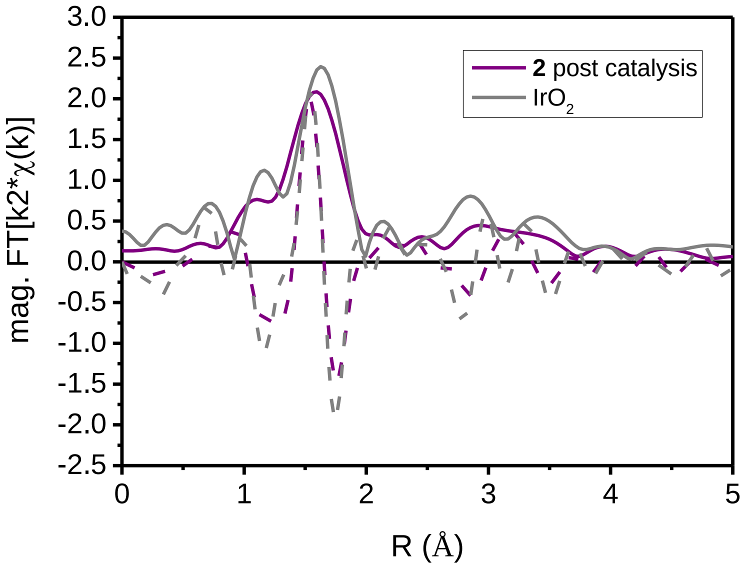

Figure S10. The magnitude (solid) and imaginary part (dashed) of the Fourier transform of the k^2-weighted EXAFS of of **2** after catalytic run and reference IrO_2.

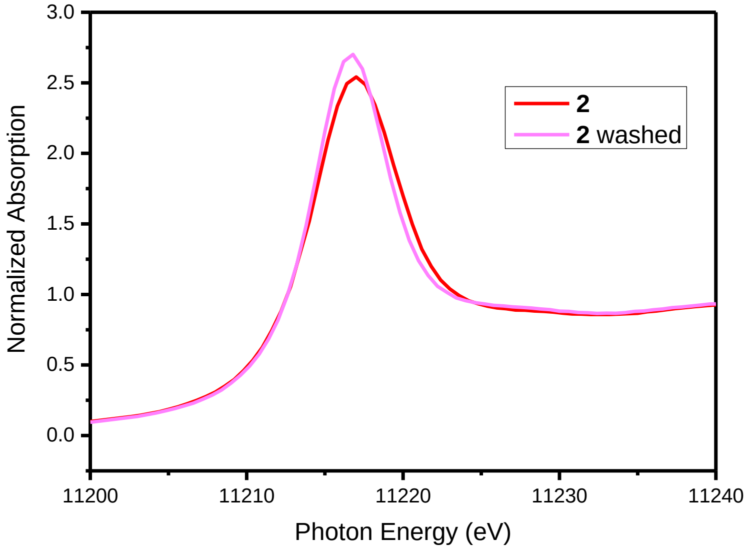

Figure S11. Ir L_3 edge XANES of **2** before and after extensive washing with water at 80°C (see caption of Figure S10 for details).

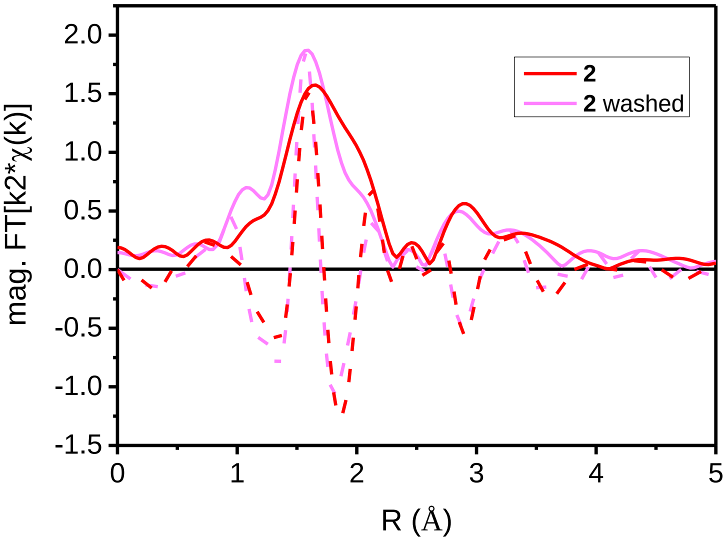

Figure S12. The magnitude (solid) and imaginary part (dashed) of the Fourier transform of the k^2-weighted EXAFS of **2** before and after extensive washing with water at 80°C. XANES Energies and EXAFS coordination parameters for **2** washed: Edge Energy (eV) = 11 214.6; S_o^2 = 0.78; CN = 5.9 ± 0.5 (Ir–O); R (Å) = 1.98 ± 0.01; σ^2 (10^{-3} Å^2) = 2.5 ± 1.1; E° (eV) = 6.3 ± 1.1; R-factor = k1: 0.003, k2: 0.006, k3: 0.009.

- **Kinetic plots for $NaIO_4$-driven WO**

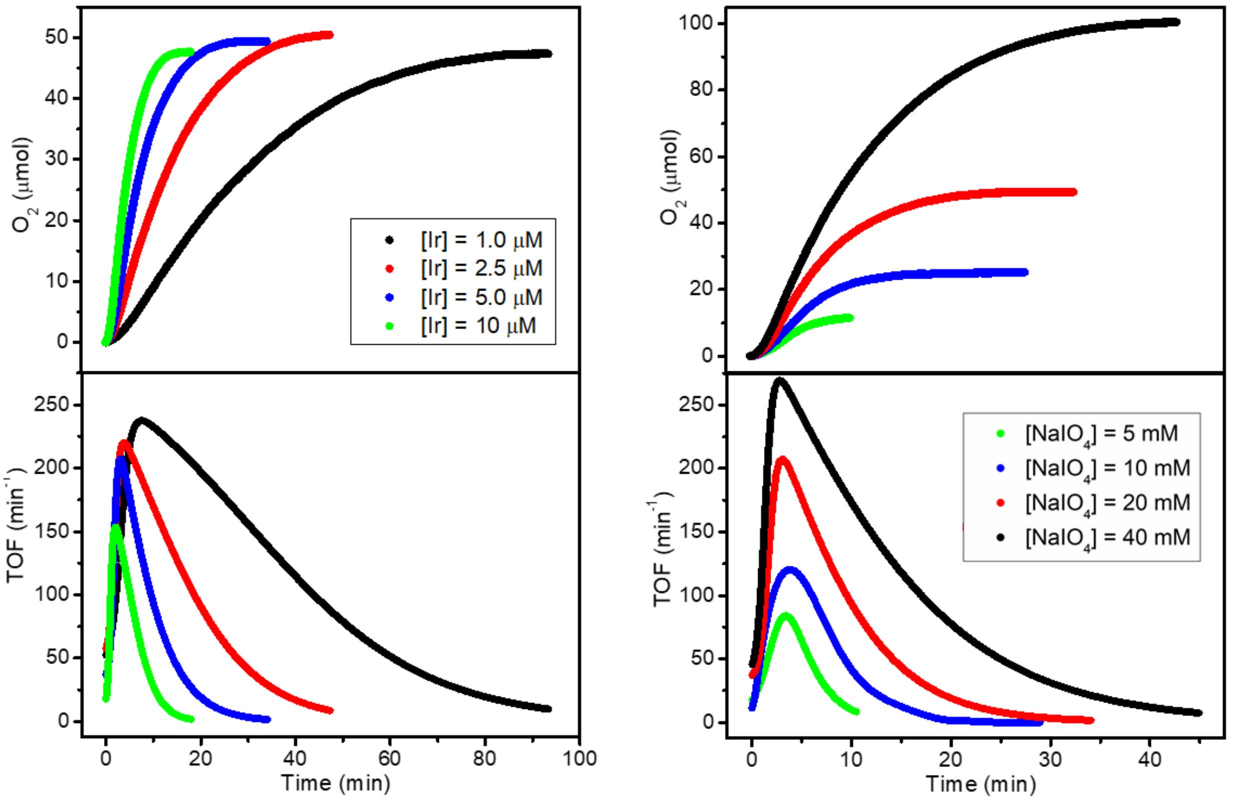

Figure S13. Kinetic profiles for WO by catalyst **1** (pH 7 by phosphate buffer, 25°C) at different catalyst (left) and $NaIO_4$ (right) concentrations.

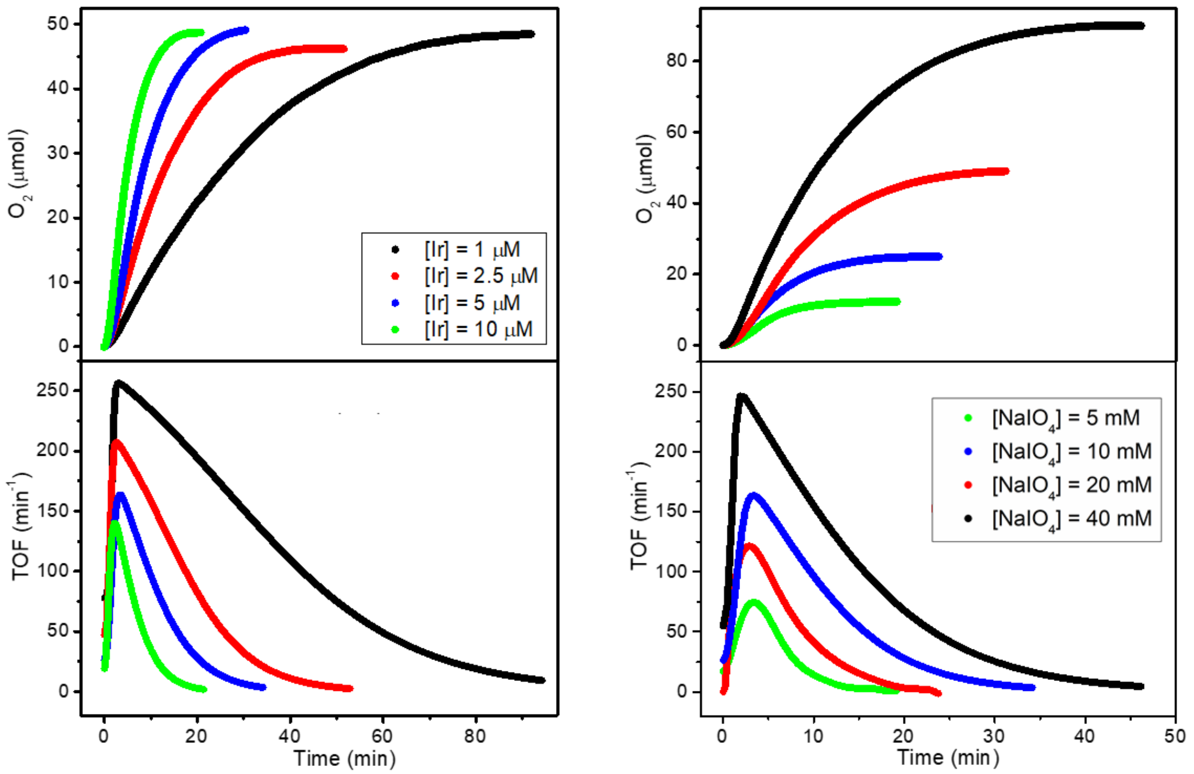

Figure S14. Kinetic profiles for WO by catalyst **2** (pH 7 by phosphate buffer, 25°C) at different catalyst (left) and $NaIO_4$ (right) concentrations.

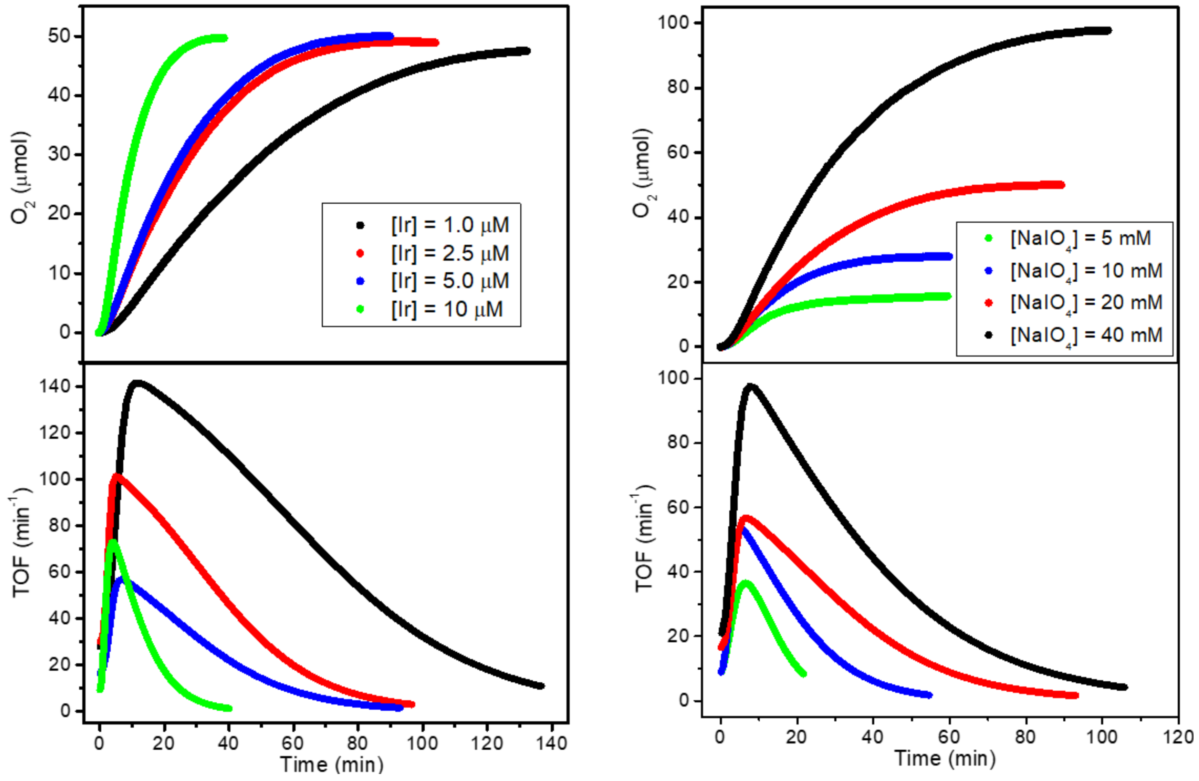

Figure S15. Kinetic profiles for WO by catalyst **4** (pH 7 by phosphate buffer, 25°C) at different catalyst (left) and $NaIO_4$ (right) concentrations.

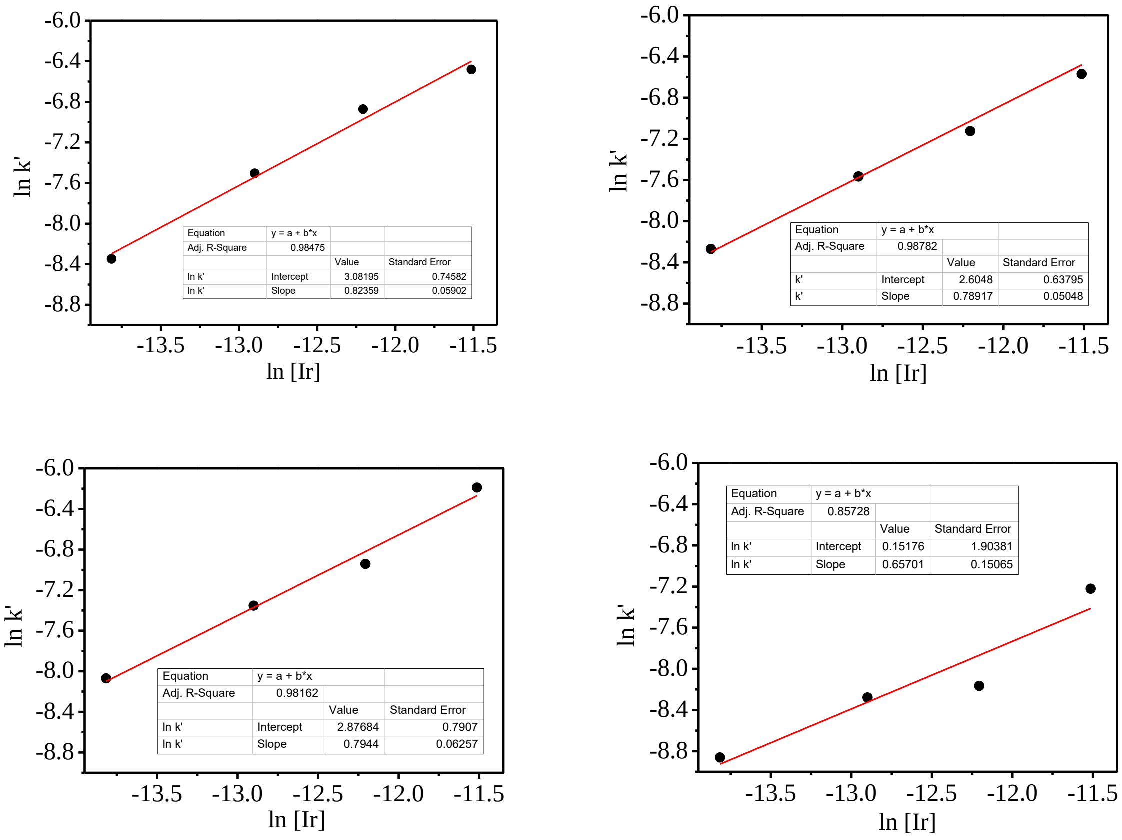

Figure S16. log k' – log [Ir] plot for catalysts **1** (up left) **2**, (up right), **3** (down left) and **4** (down right). ([$NaIO_4$] = 20 mM; [Ir] = 1.0, 2.5, 5.0, 10 μM; pH 7 by phosphate buffer).

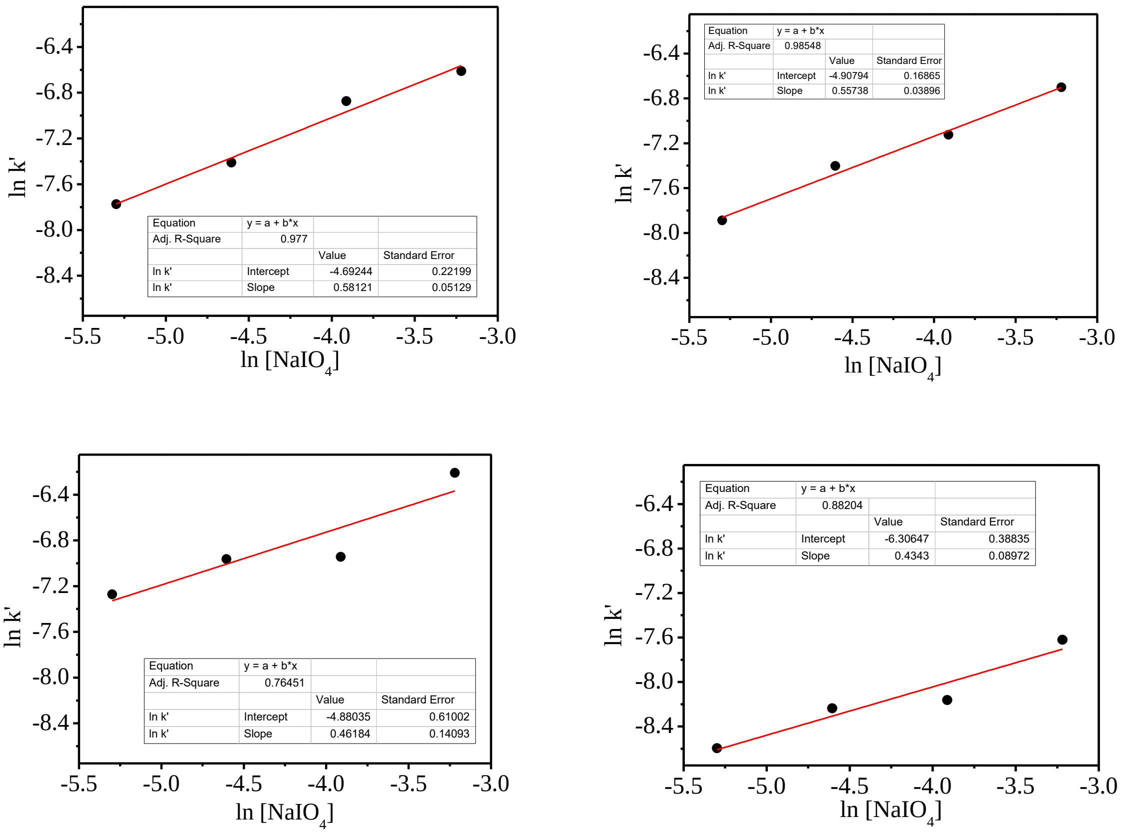

Figure S17. log k' – log [$NaIO_4$] plot for catalysts **1** (up left) **2**, (up right), **3** (down left) and **4** (down right). ([Ir] = 5 µM [$NaIO_4$] = 5.0, 10, 20, 40 mM; pH 7 by phosphate buffer).

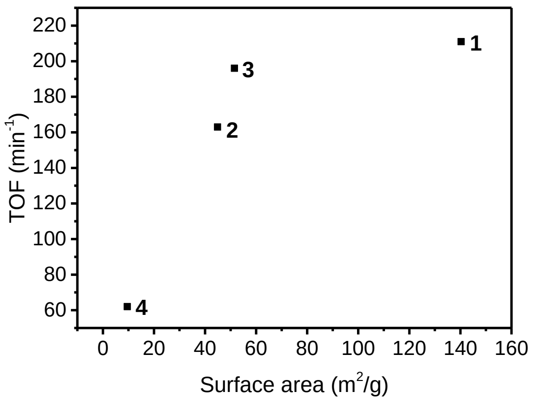

Figure S18. Comparison of TOFs ([Ir] = 5 µM, [$NaIO_4$] = 20 mM) vs. BET surface area for catalysts **1**-**4**.

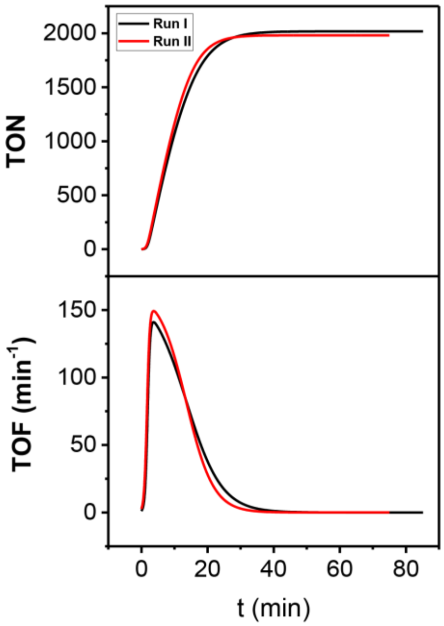

Figure S19. Comparison of kinetic profiles obtained in recyclability test for **2** ([Ir] = 5 μM, [$NaIO_4$] = 20 mM, pH 7 by phosphate buffer, 25°C). Balck trace: first catalytic run; red trace: the second catalytic run, carried out by recovering the solid catalyst used in the first experiment.
